# Genomics insights of candidiasis: mechanisms of pathogenicity and drug resistance

**DOI:** 10.3389/fmicb.2025.1531543

**Published:** 2025-02-27

**Authors:** Xin Huang, Qin Dong, Qi Zhou, Shitao Fang, Yiheng Xu, Hongjie Long, Jingyi Chen, Xiao Li, Huaguang Qin, Dan Mu, Xunchao Cai

**Affiliations:** ^1^Key Laboratory of Biodiversity Conservation and Characteristic Resource Utilization in Southwest Anhui, Anqing Forestry Technology Innovation Research Institute, School of Life Sciences, Anqing Normal University, Anqing, China; ^2^State Key Laboratory of Pharmaceutical Biotechnology, School of Life Sciences, Nanjing University, Nanjing, China; ^3^Department of Gastroenterology and Hepatology, Shenzhen University General Hospital, Shenzhen University, Shenzhen, China

**Keywords:** candidiasis, genomics, pathogenicity, drug resistance, virulence factors

## Abstract

Candidiasis, a prevalent class of human infections caused by fungi belonging to the *Candida* genus, is garnering increasing attention due to its pathogenicity and the emergence of drug resistance. The advancement of genomics technologies has offered powerful tools for investigating the pathogenic mechanisms and drug resistance characteristics of *Candida*. This comprehensive review provides an overview of the applications of genomics in candidiasis research, encompassing genome sequencing, comparative genomics, and functional genomics, along with the pathogenic features and core virulence factors of *Candida*. Moreover, this review highlights the role of genomic variations in the emergence of drug resistance, further elucidating the evolutionary and adaptive mechanisms of *Candida*. In conclusion, the review underscores the current state of research and prospective avenues for exploration of candidiasis, providing a theoretical basis for clinical treatments and public health strategies.

## 1 Introduction

**Figure 1:**
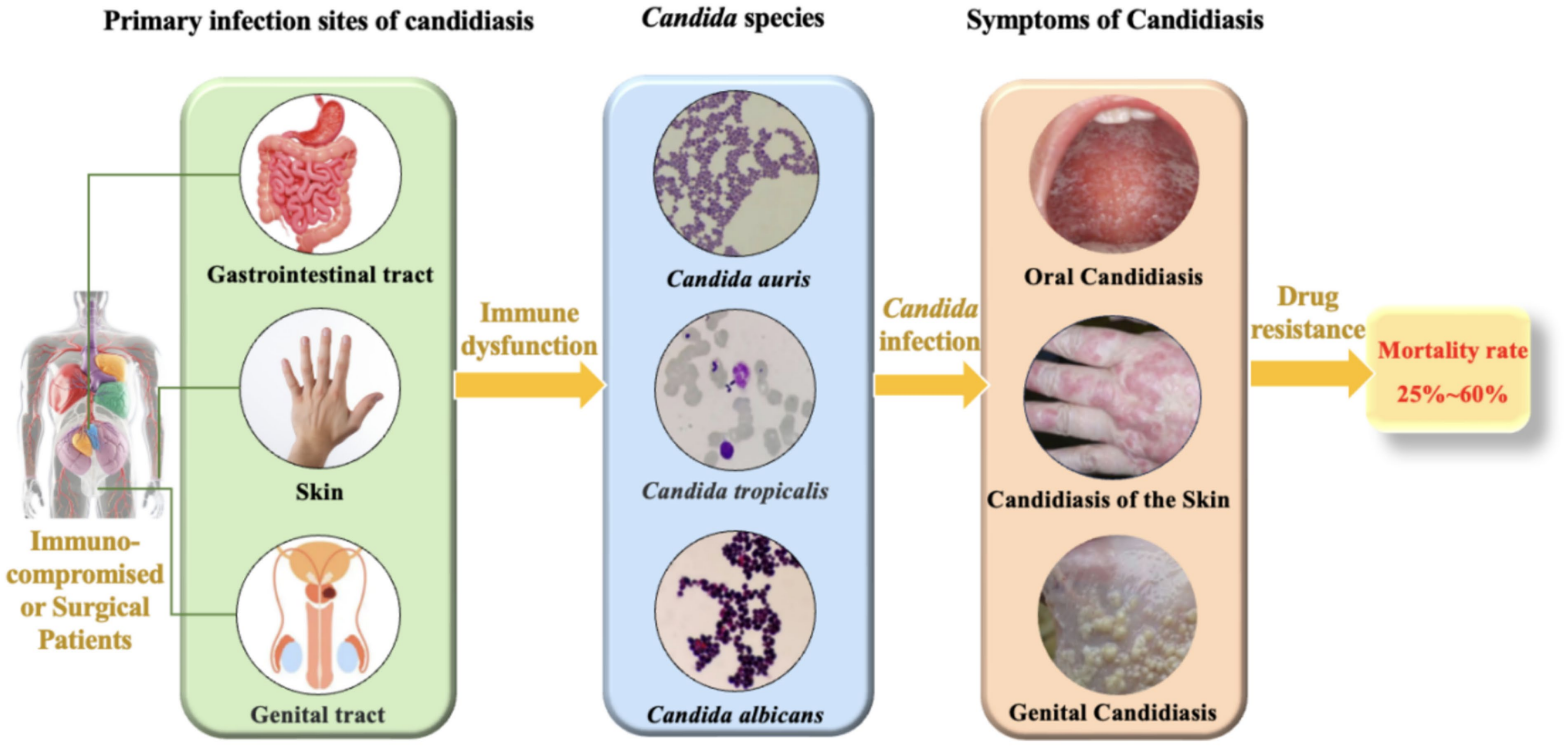
The main infection sites and mortality rates of *Candida* species.

Candidiasis is a prevalent fungal infection caused by pathogenic fungi belonging to the *Candida* genus. *Candida* is a commensal fungus and an integral member of the human normal microbiota, predominantly colonizing the skin, gastrointestinal tract, and genital tract (Shiyadeh et al., 2024). Despite their normal presence, *Candida* can cause a spectrum of infections, ranging from superficial skin and mucosal infections to invasive systemic infections (Figure 1). These infections are particularly common in immunocompromised patients, such as those with human immunodeficiency virus (HIV), organ transplant recipients, and patients undergoing chemotherapy (Bhattacharya et al., 2020; Fisher et al., 2022). Although *C. albicans* is the most prevalent pathogenic species in the genus, constituting approximately 37% of reported cases (Henriques and Williams, 2020; Marquez et al., 2023), recent studies have shown an increasing frequency of infections caused by non-albicans *Candida*. These non-albicans *Candida* species (e.g., *C. auris*, *C. krusei*, *C. tropicalis*, *C. parapsilosis*) are associated with higher mortality rates compared to *C. albicans* (Mandras et al., 2021; Husni et al., 2023) and frequently exhibit resistance to commonly used antifungal drugs (Seyoum et al., 2020; Kitaya et al., 2023), which poses significant challenges for clinical treatment. According to the 2019 Antibiotic Resistance Threats Report by the U.S. Centers for Disease Control and Prevention (CDC), over 34,000 cases and 1,700 deaths annually are attributed to drug-resistant *Candida* species (Kadri, 2020). Additionally, 323 cases of emerging multidrug-resistant *Candida* infections have been reported (Lyman et al., 2023). Given the significant impact of *Candida* infections on human health and the clinical challenges posed by antifungal resistance, *Candida* infections have become a serious threat to global public health. Consequently, a deeper understanding of the pathogenic mechanisms and resistance characteristics of *Candida* is imperative. Such knowledge is vital for devising efficacious therapeutic strategies and curbing the escalating epidemic of candidiasis.

A multitude of factors contribute to the escalating drug resistance observed in *Candida* species. The overexpression of genes encoding efflux pump proteins such as *CDR1* and *CDR2* in *C. albicans* (Rocha et al., 2017; Prasad and Rawal, 2014) and *MDR1* in *C. tropicalis* (Jin et al., 2018), facilitates the efflux of antifungal drugs like fluconazole, thereby reducing intracellular drug levels and augmenting resistance. Additionally, the ability of *Candida* to form biofilms significantly hinders drug penetration (Pereira et al., 2021). Furthermore, the prolonged use of broad-spectrum antifungal drugs, such as fluconazole and itraconazole, during hospital treatment can create selective pressure, which encourages the emergence of resistant strains in pathogens with high mutation rates (Cai et al., 2023). This can lead to the unnecessary utilization of antifungal drugs, thereby promoting resistance in patients with non-invasive candidiasis or delaying effective antifungal treatment in infected patients. Consequently, the development of drug resistance is multifaceted, encompassing genetic mechanisms such as mutations, gene expression, and alterations in cell membrane permeability (Sanglard and Odds, 2002; Czajka et al., 2023).This review endeavors to underscore the latest breakthroughs in the genomics of candidiasis, encompassing the utilization of genome sequencing, comparative genomics, and functional genomics to elucidate the pathogenic mechanisms and drug resistance. The review further expounds on the impact of genomic variations in the development of drug resistance, as well as the evolutionary and adaptive mechanisms of *Candida*, providing a theoretical basis for the clinical treatment and prevention of candidiasis.

## 2 Epidemiological trends of candidiasis and genomic response strategies

### 2.1 Epidemiological trends and basic characteristics of candidiasis

The epidemiological landscape of candidiasis is undergoing a transformation, with a global shift observed from the historically predominant *C. albicans* to drug-resistant non-albicans *Candida* species (Quindós et al., 2018). The emergence of multidrug-resistant *Candida* species, notably *C. auris*, has been identified in over 40 countries worldwide and has diverged into four distinct genetic lineages (Rhodes and Fisher, 2019), revealing their global transmission patterns and diversity. Hospital environments are pivotal in the propagation of candidiasis, where *Candida* can survive and resist routine cleaning and disinfection measures (Erganis et al., 2024). Nevertheless, the prevalence of *Candida* in special environments or workplaces, such as farms, livestock, and slaughterhouses, requires further investigation.

*Candida* species primarily inhabit the human body (Fidel, 2011), with different species predominating in various infection sites. Oral and esophageal candidiasis are mainly caused by *C. albicans*, and vaginal candidiasis is also predominantly attributed to *C. albicans*, although it can also be linked to *C. tropicalis* and *C. glabrata* (Dabas, 2013). Skin and bloodstream candidiasis can be caused by multiple *Candida* species, including *C. albicans*, *C. tropicalis*, *C. glabrata*, and *C. krusei* (Williams et al., 2013; Fidel, 2011). Additionally, *C. auris*, an emerging pathogen, has been reported to cause infections in hospital settings and is capable of colonizing the skin of patients (Proctor et al., 2021).Candidiasis poses a significant challenge to public health, with clinical studies indicating that the mortality rate associated with *Candida* infections of the bloodstream, peritoneum, and other sites is alarmingly high (Figure 1), typically ranging from 25 to 60% within 30 days of diagnosis (Rajendran et al., 2016; Hirano et al., 2015; Bassetti et al., 2015). In the United States, the incidence of invasive candidiasis is as high as about 90 cases per 100,000 people, with a rising trend in non-bloodstream infections (Ricotta et al., 2021). This situation not only increases the hospitalization burden but also leads to a significant rise in the use of antifungal drugs, exacerbating the development of antifungal resistance (Parslow and Thornton, 2022; Oliva et al., 2023). From an economic perspective, the cost of hospitalization for candidiasis is high, accompanied by longer hospital stays and higher readmission rates, further straining the healthcare system (Ricotta et al., 2021).

In clinical practice, the diagnosis of candidiasis generally relies on clinical symptoms and laboratory examination results. Candidal vaginitis, the most common form of *Candida* infection, is mainly an inflammatory change in the vagina and vulva caused by *Candida* species, especially *Candida albicans* (Jeanmonod et al., 2025). For invasive candidiasis, the diagnosis is more challenging and requires a comprehensive assessment integrating clinical manifestations and laboratory tests such as *Candida* antigen detection (Barantsevich and Barantsevich, 2022). Currently, the diagnosis is typically carried out by first evaluating the patient’s symptoms related to the suspected candidiasis site. Then, relevant laboratory tests are conducted to confirm the presence and type of *Candida*. However, there are limitations in the existing diagnostic methods. Clinicians often rely on empirical treatment, such as the overuse of antibiotics like fluconazole, potentially overlooking critical diagnostic steps, including the detection of *C. glabrata* infections (*C. glabrata* has developed a resistance rate of up to 14.3% to fluconazole and is continuously rising) and conducting drug susceptibility testing (Poves-Alvarez et al., 2019; Zheng et al., 2024), which may in turn affect the overall treatment efficacy. This can lead to misdiagnosis and inappropriate treatment (Benedict et al., 2022; Huang et al., 2023). Additionally, detecting and identifying certain *Candida* species, like *C. glabrata* and *C. parapsilosis*, present difficulties (Sobel, 2022). These challenges in diagnosis can potentially affect the treatment outcome. In summary, current diagnostic methods for candidiasis have limitations, necessitating urgent exploration of new approaches. Genomics research may revolutionize diagnosis by enhancing accuracy and timeliness.

### 2.2 Application of genomics in candidiasis research

**Figure 2:**
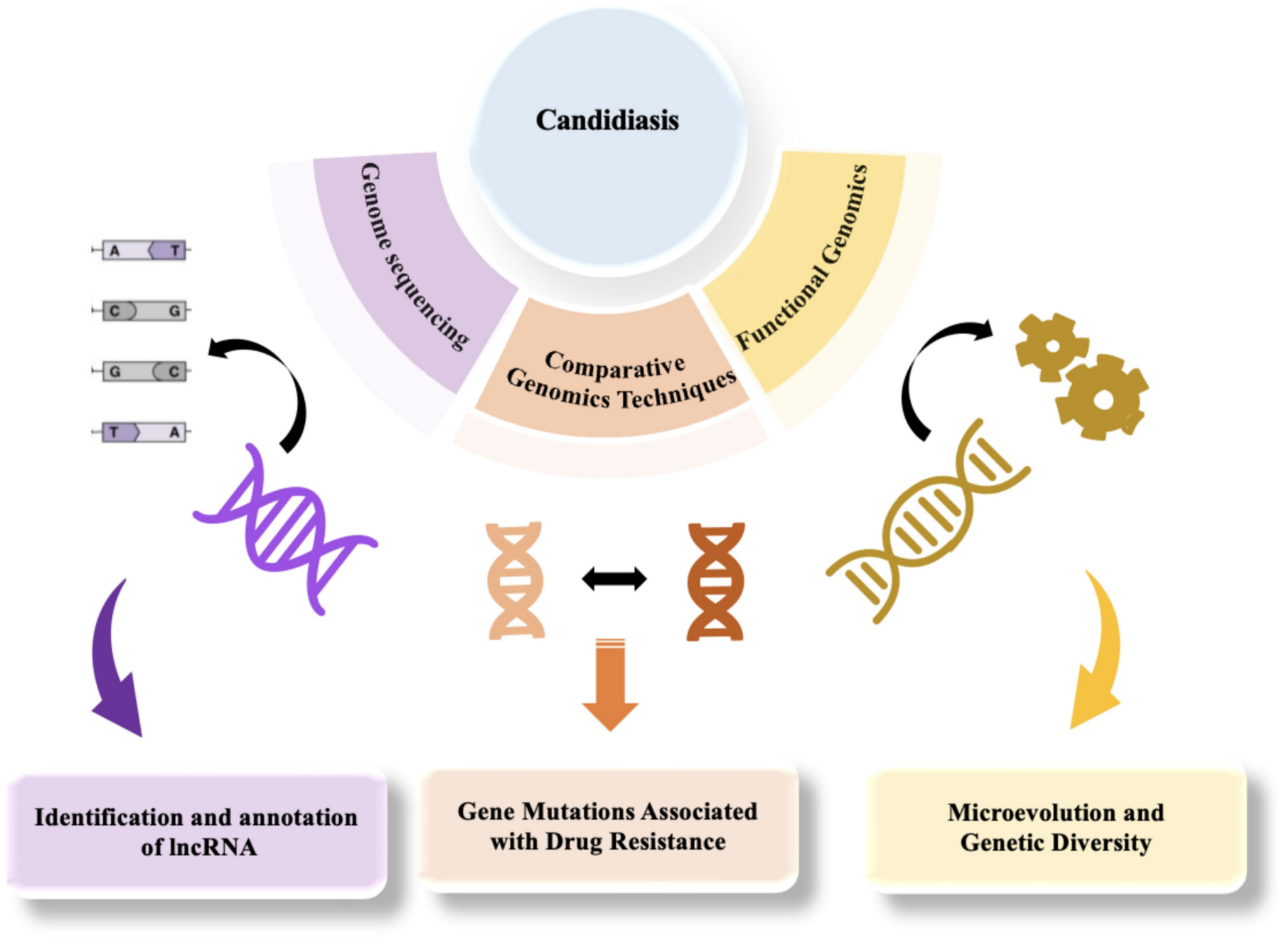
The application of genomics in the study of candidiasis, from sequencing to functional analysis.

The genome of *Candida* is primarily composed of linear chromosomes, with the specific number and size varying among species. For instance, *C. albicans* possesses eight pairs of homologous chromosomes, whereas other species such as *C. glabrata* have 13 chromosomes (Selmecki et al., 2010; Helmstetter et al., 2022; Butler et al., 2009). Generally, the *Candida* genome contains numerous repetitive sequences and transposable elements, which may play crucial roles in gene regulation and evolution (Selmecki et al., 2010; Braun et al., 2005). The *Candida* genome also includes several gene families associated with pathogenicity, such as the ALS (adhesin) family and SAP (secreted aspartyl protease) family. These genes play a crucial role in the infection process of *Candida* (Ruiz-Herrera et al., 2006; Arnaud et al., 2009; Kabir et al., 2012). Moreover, the *Candida* genome exhibits a high degree of plasticity because of genomic instability, prone to chromosomal rearrangements, gene amplifications, and horizontal gene transfers, attributes closely associated with environmental adaptation and drug resistance (Butler et al., 2009; Selmecki et al., 2010; Helmstetter et al., 2022). Comparative genomics studies have unveiled the genomic instability among various *Candida* species, such as *C. tropicalis* and *C. parapsilosis*, which are associated with their pathogenic mechanisms and ecological adaptability (Butler et al., 2009; Desjardins et al., 2011). The genomic instability of *Candida* is primarily caused by multiple internal and external factors. Firstly, the genome is rich in repetitive sequences and active transposable elements that can easily trigger homologous recombination, leading to chromosomal rearrangements, deletions, or amplifications. Meanwhile, the dynamic changes in telomeres also increase the risk of chromosome fusion or breakage (Aguilera and García-Muse, 2013). Secondly, the alternation between sexual and asexual reproduction in *Candida* increases the possibility of genomic recombination, while the emergence of aneuploidy can rapidly alter gene dosage to adapt to environmental stress (Mba et al., 2022; Marton, 2020). Furthermore, environmental stresses such as temperature, pH, nutrient deficiency, and the use of antifungal drugs can also induce genomic instability (Aguilera and Gómez-González, 2008; Pais et al., 2019; Mba et al., 2022). Additionally, *Candida* can acquire exogenous genes from other microorganisms through horizontal gene transfer, further altering the genomic structure and acquiring new functions (Bezerra et al., 2013). Finally, defects in DNA repair mechanisms exacerbate genomic instability (Aguilera and Gómez-González, 2008). These factors collectively enable *Candida* to rapidly respond to environmental stress, enhance pathogenicity, and become a significant driving force for its environmental adaptation and evolution (Pais et al., 2019).Candidiasis poses a significant global health challenge, with the increasing prevalence of multidrug resistance and the emergence of novel pathogens complicating therapeutic interventions. Traditional research methods have limitations in accurately identifying *Candida* species, elucidating resistance mechanisms, and facilitating rapid diagnostics. In this context, genomic technologies have emerged as pivotal tools in the study of candidiasis, particularly through genome sequencing, comparative genomics, and functional genomics, which enable researchers to gain a deeper understanding of the pathogenic mechanisms and resistance characteristics of *Candida* (Figure 2). In recent years, significant progress has been made in the field of genomics in the study of candidiasis, allowing researchers to analyze the genetic features of *Candida* in detail and elucidate the complex relationships between its genetics, pathogenicity, and resistance. For example, Schikora-Tamarit and Gabaldón (2022) emphasized the central role of genomic sequencing studies in revealing the pathogenic mechanisms of *Candida*, providing important guidance for research in this field (Schikora-Tamarit and Gabaldón, 2022). Recently, Mathur et al. (2024) made new breakthroughs in this area. They utilized high-throughput genomic sequencing and bioinformatics analysis to successfully identify long non-coding RNAs (lncRNAs) in *C. auris*. These lncRNAs exhibit a distinct chromosomal distribution, with GC content concentrated between 40 and 45%, and most are predicted to be localized in the nucleus (Mathur et al., 2024). These findings suggest that lncRNAs may play important roles in regulating gene expression and maintaining genome stability. Furthermore, Mathur et al. (2024) observed differential expression patterns of lncRNAs at different infection stages, which could be intricately linked to the pathogenicity and drug resistance of *C. auris*, shedding new light on *Candida*’s pathogenic mechanisms. Additionally, through protein interaction network analysis, the researchers revealed potential interactions between lncRNAs and proteins, offering scientific evidence and potential targets for further exploring the development of drug resistance in *Candida* and the development of new therapies.

In terms of drug resistance, researchers have identified multiple gene mutations and mechanisms associated with resistance. Kannan et al. (2019) discovered 18 non-synonymous single nucleotide polymorphisms (SNPs) associated with multidrug resistance through comparative genomic analysis, with six SNPs being particularly implicated in resistance to various antifungal agents (Kannan et al., 2019). These findings indicate that genomic analysis not only provides clues to resistance mechanisms but also helps identify specific resistance genes. Czajka et al. (2023) summarized the antifungal drug resistance mechanisms of *Candida* species, pointing out that the development of resistance is primarily attributed to three factors: gene mutations, transcriptional regulation, and biofilm formation (Czajka et al., 2023). Moreover, Helmstetter et al. (2022) conducted an in-depth investigation into the microevolution of *C. glabrata* in clinical settings, revealing high levels of variation and recombination in mitochondrial genomes, pathogenic genes, and drug targets. This not only highlights the significant genetic diversity of *C. glabrata* but also demonstrates its capacity for rapid adaptability. It emphasizes that the inherent genetic diversity within *C. glabrata* populations may have a significant impact on its pathogenicity and drug resistance, with microevolution and genetic exchange/recombination being the two key primary drivers of this diversity (Helmstetter et al., 2022). Chow et al. (2020) traced the global spread history of the multidrug-resistant *C. auris* by comparing the genomes of 304 isolates, revealing its population structure and evolutionary relationships across different geographic regions (Chow et al., 2020). The results corroborate the hypothesis that *C. auris* may have spread to different countries through multiple independent introductions and provide insights into its global transmission patterns, which encompass multiple origins, geographic admixture, rapid evolutionary changes, and the maintenance of population structure. These findings are pivotal for public health surveillance, hospital infection control, and the development of personalized treatment strategies. However, the research did not extensively discuss the drug resistance characteristics of different lineages of *C. auris* and their relationship with genomic variations, thus necessitating the application of comparative genomics and further exploration.

Comparative genomics allows scientists to systematically compare the genomes of different *Candida* species, thereby identifying specific genes associated with pathogenicity and drug resistance. For example, Kannan et al. (2019) utilized comparative genomics to elucidate the genetic basis of multidrug resistance (MDR) mechanisms within the *Candida* genus, demonstrating that genomic analysis can identify important mutations (e.g., *MRR1*, *FKS1*, *ERG3*, and *ERG4* gene) related to drug resistance (Kannan et al., 2019). Additionally, Maphanga et al. (2021) revealed the genetic basis of multidrug resistance in *C. auris* isolates from bloodstream infections through genomic analysis, identifying a suite of gene mutations associated with antifungal drug resistance, including *ERG11*, *FKS1*, *UPC2*, and *FKS2* (Maphanga et al., 2021). These genes are also widely present in other *Candida* species (such as *C. albicans*, *C. glabrata*, and *C. parapsilosis*.), constituting a critical genetic basis for the multidrug resistance of *Candida* (Ibe et al., 2025; Boyce, 2023; Bédard et al., 2024; ElFeky et al., 2025). This provides new insights into the mechanisms of antifungal resistance in *Candida* and offers important reference data for clinical antifungal therapy.

In terms of virulence and resistance genes, significant differences and similarities exist among *Candida* species. For instance, in *C. albicans*, virulence is primarily regulated by adhesin genes (ALS family), hydrolase genes (SAP family), and morphogenesis-related genes such as *EFG1* and *CPH1* (Davari et al., 2019; Bras et al., 2024). In contrast, virulence of *C. glabrata* relies on the EPA family of adhesin genes and drug resistance-related genes such as *CDR1* and *PDH1* (Caudle et al., 2011). Notably, *C. tropicalis* and *C. krusei* exhibit unique virulence and resistance mechanisms, with *C. tropicalis* depending on the SAP family, which degrades host proteins to facilitate invasion and evasion of the host immune response, and also enables nutrient acquisition from the host, and *BCR1* genes, which regulate cell wall remodeling, promote hyphal formation, assist *Candida* in adapting to different environments, resist host immune responses, and invade host tissues to initiate infection, while *C. krusei* demonstrates intrinsic resistance to fluconazole, potentially linked to mutations in the *ERG11* gene (Pais et al., 2016; Modrzewska and Kurnatowski, 2015). Despite these differences, conserved mechanisms such as efflux pump genes and biofilm formation genes are shared across *Candida* species (Frías-De-León et al., 2021). However, variations in genome size and structure, gene family expansion, horizontal gene transfer, and specific virulence or resistance mechanisms highlight the diversity within the *Candida* genus (Zhou et al., 2021).

While the above studies have identified drug resistance-related gene mutations in pathogenic fungi at the genomic level, they lack further functional validation and mechanistic investigation, which limits a deeper understanding of resistance mechanisms. Consequently, there is an imperative for comprehensive research in functional genomics to elucidate the adaptive significance of these genes across different *Candida* species.

In the study of *Candida* functional genomics, to comprehensively explore the molecular basis of *Candida* drug resistance and pathogenic mechanisms, it is necessary to adopt various experimental methods for the validation of gene functions. Firstly, gene function validation experiments are one of the core methodologies. Through gene knockout or overexpression experiments, we can directly verify the roles that specific genes play in drug resistance and virulence of *Candida* species, such as *C. glabrata* and *C. tropicalis* (Gale et al., 2023; Paul et al., 2022). This method provides us with intuitive evidence demonstrating the impact of gene function on the biological characteristics of *Candida*. Secondly, expression analysis is also an indispensable part. Advanced technologies such as RNA sequencing can be utilized to deeply analyze the gene expression changes of *Candida* under different environmental conditions (e.g., antifungal drug treatment; Zuber et al., 2023; Helmstetter et al., 2022). By comparing the gene expression differences between resistant strains and sensitive strains, key genes related to drug resistance can be identified, providing important clues for subsequent research. Moreover, the application of genome sequencing technology has greatly advanced the development of *Candida* functional genomics. Through whole-genome sequencing, we are able to unveil the genetic variations between resistant and susceptible strains of *Candida*, which may involve critical aspects of the resistance mechanisms (Helmstetter et al., 2022; Burrack et al., 2022). Based on these findings, we can further explore the molecular mechanisms of drug resistance, providing a theoretical foundation for the development of novel antifungal agents. Finally, functional genomics analysis, as a systematic research approach, integrates data from multiple aspects including gene expression and genetic variation (Schikora-Tamarit and Gabaldón, 2022; Helmstetter et al., 2022; Burrack et al., 2022). Using this method, a more comprehensive understanding can be gained of how *Candida* regulates its genome to adapt to the host environment during the infection process. These comprehensive verifications and analyses not only contribute to unveiling the complex mechanisms of *Candida* resistance but also provide a scientific basis for formulating effective treatment regimens.

In functional genomics, researchers have explored how *Candida* species regulates its genome to adapt to the host environment during infection by analyzing gene expression and gene function. Schikora-Tamarit and Gabaldón (2022) highlighted that the study of genomic sequences is fundamental for identifying pathogenic and drug-resistant characteristics, emphasizing the importance of comparative genomics and population genomics in elucidating these traits (Schikora-Tamarit and Gabaldón, 2022). Building on the foundation of comparative genomics, studies using improved annotation tools, CRISPR-Cas9 technology, and long-read sequencing have shown that genomic alterations, such as gene expansion and horizontal gene transfer, are intimately linked to the emergence of fungal pathogenicity and drug resistance. Population genomics research has revealed the genetic structure of fungal pathogen populations, identifying genetic variants associated with these traits through genome-wide association studies. Moreover, directed evolution experiments have detected phenotypic mutations under controlled conditions, further simplifying the identification of mutations related to pathogenicity and drug resistance (Schikora-Tamarit and Gabaldón, 2022). Other studies, such as the *Candida* Comparative Genomics Project that sequenced and annotated multiple *Candida* species including *C. albicans* (WO-1), *C. tropicalis*, *C. guilliermondii*, and *C. lusitaniae* (Butler et al., 2009), population genomics research on *C. albicans* revealing evidence of gene flow and (para) sexuality in nature (Ropars et al., 2018), recent investigations on gene selection and drug resistance analyzing the genomes and phenotypes of 2,000 *Candida* strains (Schikora-Tamarit and Gabaldón, 2024), and studies utilizing direct RNA sequencing technology to examine the mitochondrial transcriptome of *C. albicans* (Piątkowski et al., 2024), have also made significant contributions to the understanding of this field.

In addition to direct whole genome sequencing for comparative genomics, transposon mutagenesis and transposon insertion site sequencing (Tn-seq) technologies play crucial roles in understanding *Candida*’s pathogenic mechanisms. Transposon mutagenesis technology is not only applicable for large-scale screening of genes affecting *Candida* virulence and identification of virulence factors but also for investigating the functions of genes that play a pivotal role during infection processes, thereby uncovering the functions of previously unknown genes and deepening our understanding of *Candida* biology (Kim et al., 2024; Zhou et al., 2024). Moreover, Tn-seq facilitates precise localization of transposon insertion sites, identifies affected genes, and promotes accurate studies of gene function, aiding functional genomics research to comprehensively elucidate the roles of genes in *Candida* virulence and adaptability (Nickels et al., 2024; Xiong et al., 2024). For instance, utilizing Tn-seq technology, researchers have identified multiple known virulence factors and novel genes critical for virulence and adaptability across the entire genome of *C. glabrata*, providing new insights into its pathogenic mechanisms (Nickels et al., 2024). Researchers have also employed functional genomics analysis using transposon mutant libraries to study the adaptability of *C. albicans* under various environmental conditions, discovering several important genes related to adaptability, including novel genes involved in cell wall synthesis, metabolism, and stress response (Xiong et al., 2024).

These above studies contribute significantly to understanding candidiasis by elucidating how genetic variations and mutations in *Candida* species affect their adaptability to host environments, virulence, and resistance to antifungal treatments, and by providing a solid foundation for the development of targeted therapeutic strategies and personalized treatment regimens (Jacobs et al., 2024; Schikora-Tamarit and Gabaldón, 2024).

In the study of candidiasis, to optimize treatment strategies and facilitate the creation of innovative antifungal drugs and diagnostic technologies, we should pay more attention to the following comparative genomics and functional genomics techniques. In the field of comparative genomics technology, noteworthy aspects include genome sequencing and alignment, evolutionary analysis, and genome collinearity analysis. These techniques can unveil the genomic diversity, evolutionary relationships, and structural similarities of *Candida* genomes, providing crucial information for understanding the adaptability and pathogenicity of *Candida*. Specifically, genome sequencing and alignment technologies can identify single nucleotide polymorphisms (SNPs), insertions/deletions (InDels), and structural variations (SVs) (Arafa et al., 2023). Evolutionary analysis aids in elucidating the evolutionary history of *Candida* and the mechanisms underlying the evolution of traits such as drug resistance (Morschhäuser, 2024). Meanwhile, genome collinearity analysis can detect events such as genome rearrangements and gene duplications (Arafa et al., 2023), which are closely related to the adaptability and pathogenicity of *Candida*. In the field of functional genomics technology, transcriptomics, gene knockout and knock-in technologies, as well as proteomics and metabolomics, all hold significant value. Transcriptomics can identify differentially expressed genes of *Candida* under various conditions (Xiong et al., 2024), providing clues for drug target prediction (Xiong et al., 2024). Gene knockout and knock-in technologies (e.g., CRISPR-Cas9) can directly elucidate the functions of specific genes in *Candida* growth, pathogenicity, and drug resistance, while also identifying potential drug targets (Wang et al., 2022). Proteomics and metabolomics further unveil the expression and regulatory mechanisms of functional genes in *Candida* (Xiong et al., 2024). In the study of candidiasis, these aforementioned techniques each have their own characteristics and complement each other. For instance, genome sequencing and alignment can pinpoint specific genetic variations that may confer drug resistance or enhance virulence (Gaston et al., 2025), while transcriptomics can reveal how these genetic changes impact gene expression patterns under different environmental conditions (Zhang et al., 2025). Gene knockout and knock-in technologies allow for the functional validation of these genes, confirming their roles in pathogenicity and drug resistance (Hou et al., 2025). Meanwhile, proteomics and metabolomics provide insights into the downstream effects of these genetic and transcriptional changes on cellular processes and metabolic pathways. Therefore, the research on candidiasis should comprehensively integrate these technologies to jointly promote the optimization of treatment strategies and the development of innovative antifungal drugs and diagnostic techniques.

In summary, the application of genomics technologies has markedly advanced in-depth research on candidiasis, providing a theoretical framework for clinical management and guiding the development of future antifungal drug development (Figure 2). However, considering the global challenge of candidiasis, it is still necessary to delve into the molecular mechanisms and evolutionary pathways of drug resistance. This exploration should be complemented by optimizing treatment strategies through comparative and functional genomics, as well as by fostering the creation of innovative antifungal drugs and diagnostic techniques. Through interdisciplinary collaboration and ongoing innovation, both greater breakthroughs in the research and treatment of candidiasis are anticipated.

## 3 Pathogenic mechanisms of *Candida*

### 3.1 Pathogenic characteristics of *Candida* species

**Figure 3:**
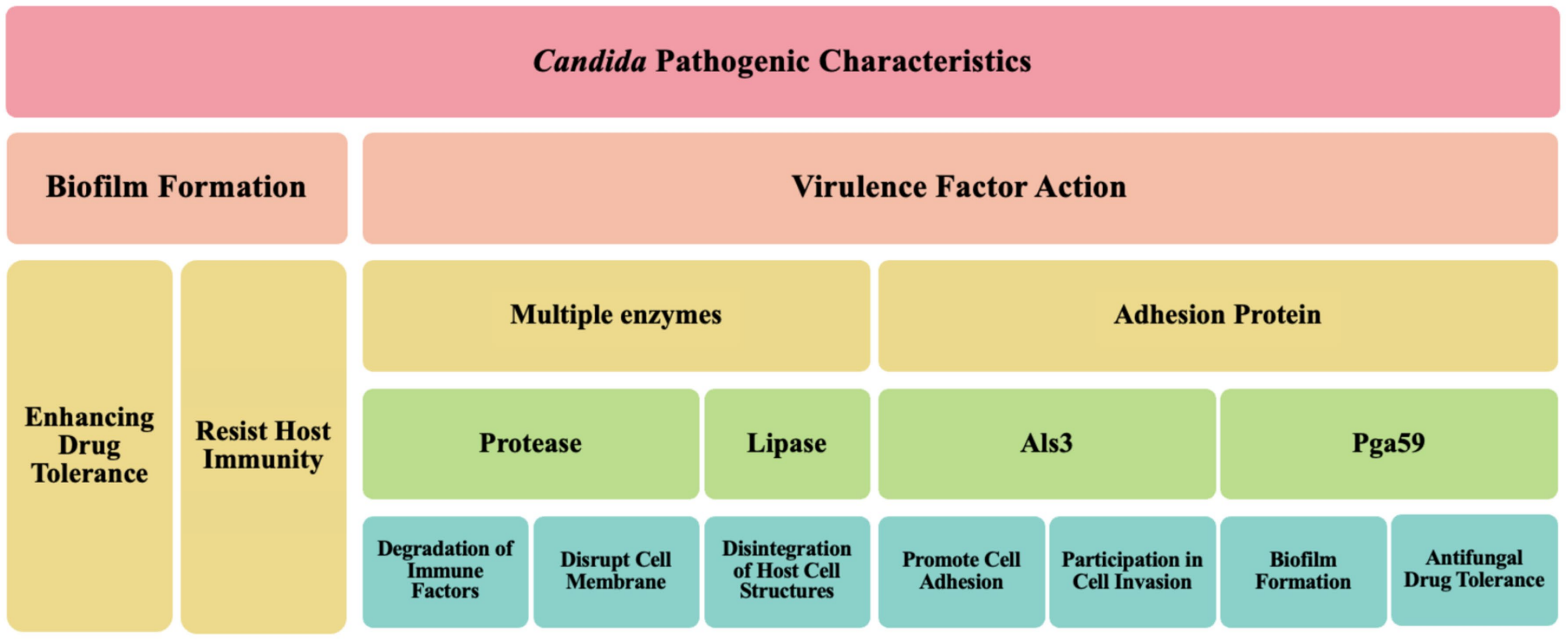
Key factors of *Candida* pathogenic mechanisms.

*Candida*, a significant human pathogenic fungus, its pathogenic characteristics are primarily manifested in biofilm formation and the action of virulence factors (Figure 3). Biofilm formation is a key feature of *Candida* infections, significantly enhancing its tolerance to antifungal drugs. These biofilms are typically complex structures composed of fungal cells, extracellular matrix, and other microbial symbionts, forming a protective barrier that shields *Candida* from both the host’s immune response and antifungal treatment attacks (Gómez-Gaviria and Mora-Montes, 2020). Further research, such as that revealed by Kumar and Kumar (2024), indicates that the biofilm formation mechanism in *C. albicans* involves multiple molecular determinants, including the precise regulation by transcription factors (e.g., CaNdt80p, Bcr1, and Efg1) and regulatory factors (e.g., Ras1-Cdc35, Cyr1, and Stt4-Mss4-Sfk1). These factors work in concert to control the expression of genes related to adhesion, hyphal/pseudohyphal growth, persister cell transformation, and overall biofilm formation. Notably, Cyr1, as an adenylate cyclase, plays a central role in the Ras/cAMP signaling pathway, catalyzing the production of cAMP, which serves as a crucial second messenger in regulating hyphal growth and various other cellular responses. Additionally, the Ras1 protein, a key component of the Ras/cAMP signaling pathway, not only participates in the regulation of cell shape but also exerts precise control over various stress responses, which is essential for nutrient sensing and uptake in *C. albicans*. Moreover, Cdc35, another adenylate cyclase, plays a significant role in the regulation of hyphal growth in *C. albicans*. The complex gene expression differences and regulation within the biofilm not only lead to adaptive changes in the morphology and metabolism of *Candida* but also enable it to form a robust protective barrier composed of fungal cells, an extracellular matrix, and other microbial symbionts. This barrier effectively resists attacks from the host immune system and exhibits significant resistance to antifungal treatments (Kumar and Kumar, 2024). Furthermore, previous studies have indicated that *Candida* promotes its colonization and infection capabilities by forming biofilms on host tissues and medical implants (Villard et al., 2015; Shinde et al., 2012; Gao et al., 2013), corroborating the latest findings on *C. albicans* biofilm formation mechanisms by Kumar and Kumar (2024). This highlights the critical role and complexity of *Candida* biofilms in their pathogenic processes. According to a recent study by Zeng et al. (2023), Rubiginosin C can effectively inhibit the biofilm formation and morphological transitions of *C. auris* and *C. albicans*, thereby exerting an inhibitory effect without causing lethal effects on host cells (Zeng et al., 2023). This finding underscores the importance of biofilms in the challenges of antifungal therapy and provides strong support for the development of novel antifungal drugs.

The pathogenicity of *Candida* is closely related to its multiple virulence factors. On one hand, *Candida* can produce various enzymes, such as proteases and lipases, which are instrumental in the invasion and damage of host tissues. For example, Lin et al. (2024) demonstrated that *C. albicans* can secrete candidalysin, a protease or cytolytic peptide toxin, which targets the sulfated glycosaminoglycans of host epithelial cells (Lin et al., 2024). This indicates that *Candida* can degrade host cell structures by releasing enzymes to obtain nutrients and enhance its survival capabilities (Bhattacharya et al., 2020). On the other hand, the adhesins on the surface of *Candida* also significantly influence its pathogenicity. These adhesins, such as Als3, are multifunctional, not only promoting cell adhesion but also participating in cell invasion, thereby helping *Candida* effectively attach to host cells (Baillie and Douglas, 1999). Additionally, these adhesins can mediate the translocation of *Candida* across the intestinal barrier, leading to systemic infections (Chong et al., 2018; Mochon et al., 2010).

Collectively, the pathogenic characteristics of *Candida* are primarily achieved through the formation of biofilms and the action of multiple virulence factors (Table 1), which enable it to successfully survive and cause infections in various host environments.

**Table 1:** The virulence factors of *Candida* and their functions.

Virulence factors	Function	References
Biofilm formation (ALS family proteins and Pga 59)	Enhances tolerance to antifungal drugs; forms a protective barrier against host immune response and antifungal treatments.	Gómez-Gaviria and Mora-Montes (2020) and Kumar and Kumar (2024)
Transcription factors (CaNdt80p, Bcr1, Efg1)	Regulate genes related to adhesion, hyphal/pseudohyphal growth, persister cell transformation, and biofilm formation.	Kumar and Kumar (2024)
Ras1-Cdc35, Cyr1, and Stt4-Mss4-Sfk1	Regulatory factors controlling biofilm formation through signaling pathways.	Kumar and Kumar (2024)
Cyr1 (Adenylate Cyclase)	Central role in Ras/cAMP signaling pathway; catalyzes cAMP production, regulating hyphal growth and cellular responses.	Kumar and Kumar (2024)
Ras1 protein	Participates in cell shape regulation and stress responses; essential for nutrient sensing and uptake.	Kumar and Kumar (2024)
Cdc35 (Adenylate Cyclase)	Significant role in the regulation of hyphal growth in *C. albicans*.	Kumar and Kumar (2024)
Candidalysin	A protease or cytolytic peptide toxin that targets sulfated glycosaminoglycans of host epithelial cells.	Lin et al. (2024)
Proteases and Lipases	Degrade host cell structures to obtain nutrients and enhance survival capabilities.	Bhattacharya et al. (2020)
Adhesins (e.g., Als3)	Promote cell adhesion and invasion; mediate translocation across the intestinal barrier, leading to systemic infections.	Baillie and Douglas (1999), Chong et al. (2018), and Mochon et al. (2010)

In the pathogenic mechanisms of *Candida*, core virulence factors (Table 1) play a vital role. Key virulence factors encompass a spectrum of enzymes and adhesion proteins that synergistically target the host to facilitate infection onset and progression.

#### 3.1.1 Crucial factors in biofilm formation and pathogenicity of *Candida*

*Candida* species, especially *C. albicans*, are significant pathogens causing diverse infections. Their ability to form drug- and immune-resistant biofilms is crucial for pathogenicity, necessitating an understanding of biofilm formation mechanisms for effective treatment.

Adhesins of *Candida* are also central to their pathogenicity, binding to specific receptors on the surface of host cells to promote adhesion and colonization. Studies have shown that adhesins such as the ALS family proteins not only enhance the adhesion of *Candida* to epithelial cells (Muñoz et al., 2018; Nishimoto et al., 2020), but also participate in biofilm formation, significantly increasing its survival and drug resistance within biofilms. These biofilms are closely associated with antifungal drug tolerance and host immune evasion, making them a major challenge in clinical settings.

In addition to the ALS family proteins, another key component in biofilm formation is Pga59. Pga59 is an amyloid protein in the cell wall of *C. albicans*, and X-ray diffraction measurements have revealed its critical role in adhesion and biofilm establishment (Mourer et al., 2023). Given that biofilms are closely associated with antifungal drug tolerance and host immune evasion, Pga59 plays a significant role in the pathogenic mechanisms of *C. albicans*, with recent studies highlighting its major contribution to biofilm structural stability (Mourer et al., 2023).

In summary, the adhesins of *Candida*, particularly the ALS family proteins and Pga59, are key in biofilm formation and pathogenicity, enhancing adhesion, stability, and drug resistance. Future research may offer new strategies to combat *Candida* infections.

#### 3.1.2 Other important virulence factors of *Candida*

*Candida* proteases, such as the SAP family proteases, act as virulence factors by effectively degrading host immune factors and extracellular matrix components, thereby enabling the fungus to evade host immune surveillance and promote its invasiveness (Nishimoto et al., 2020). Specifically, the Sap2 protease not only degrades host immunoglobulins but also promotes fungal growth by compromising the integrity of host cell membranes. Additionally, recent studies have confirmed the critical role of Sap2 in immune evasion (Lin et al., 2023).

Additionally, the virulence factors of *Candida* also include specific cell wall components (e.g., *β*-glucan). These components provide structural stability and modulate immune responses by interacting with the host’s immune system, thereby promoting the pathogenicity of *Candida* (Yoo et al., 2020). *β*-glucan can be recognized by the Dectin-1 receptor on the surface of host cells, thereby activating the host’s immune response (Netea et al., 2006). This process not only inhibits osteoclast-mediated bone resorption (Zhu et al., 2017) but also suppresses inflammation and bone destruction by producing calcitonin gene-related peptide (CGRP) (Maruyama et al., 2017). These findings also explain the phenomenon previously observed by Amanai et al. (2008), where Candida-infected rats in an experimental osteoarthritis model exhibited irregular new bone formation and significant bone resorption, leading to severe joint bone deformities (Amanai et al., 2008). Therefore, specific cell wall components of *Candida* can significantly augment its pathogenicity by influencing host immune response and bone resorption processes.

In summary, the core virulence factors of *Candida*, including various enzymes and adhesion proteins, significantly augment its pathogenicity by disrupting host cells and their immune responses, making it a dangerous pathogen.

## 4 Mechanisms of antifungal resistance in *Candida*

### 4.1 Overview of major drug resistance types and their mechanisms

**Figure 4:**
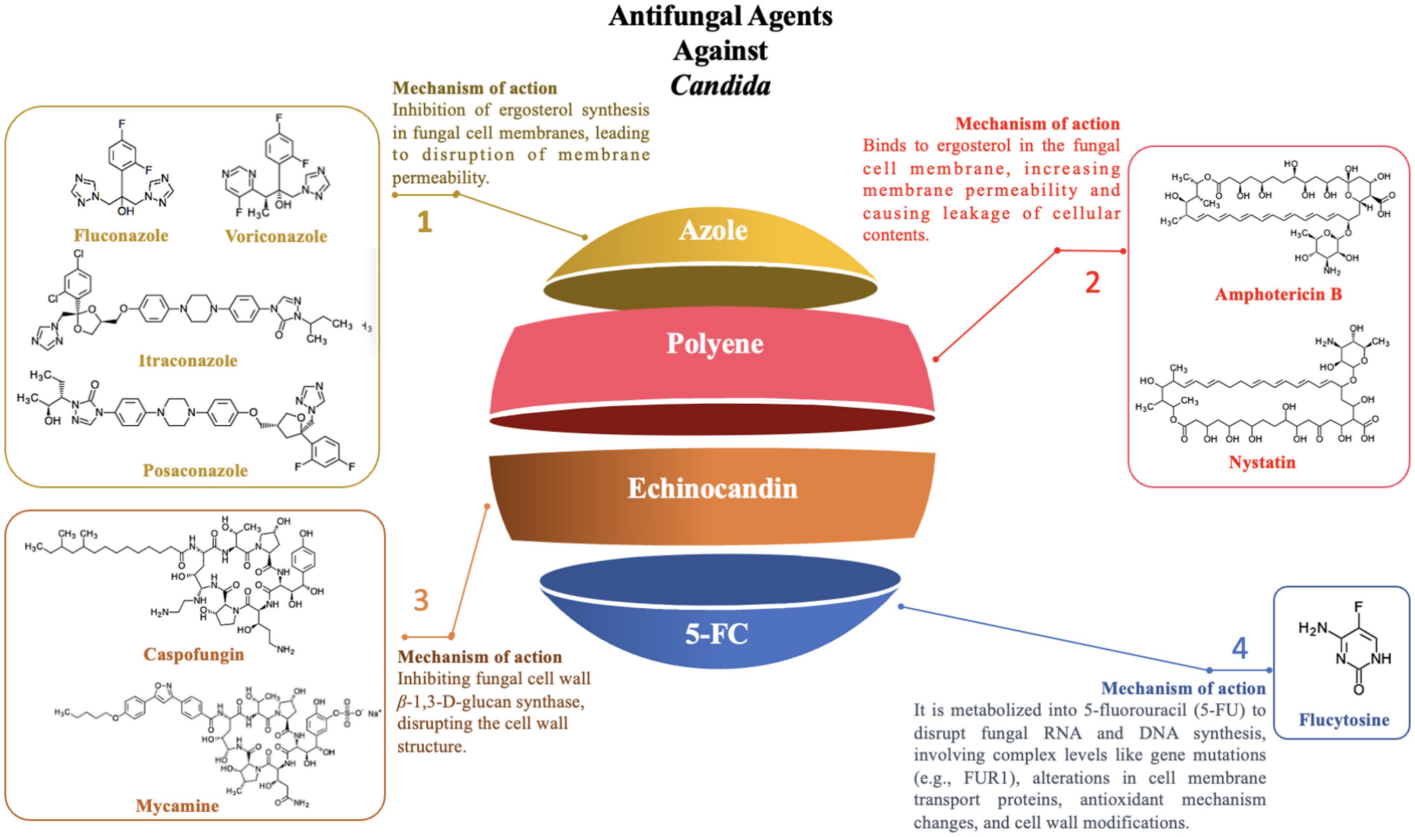
Antifungal agents against *Candida.*

With the widespread use of antifungal agents, antifungal resistance in *Candida* species has become an increasingly severe issue. This resistance issue is mediated by a variety of mechanisms that are species-specific and can be categorized as follows (Figure 4):

#### 4.1.1 Azole resistance

Azole drugs, including fluconazole, itraconazole, posaconazole, and voriconazole, primarily target the sterol-14-*α*-demethylase (CYP51) enzyme, which is pivotal in ergosterol synthesis, thereby disrupting the formation of the fungal cell membrane. Among these, the mechanism of fluconazole resistance in *Candida* primarily involves mutations in the *ERG11* gene and the overexpression of specific transporters (e.g., *CDR1* and *CDR2*). The *ERG11* gene encodes *CYP51* in *Candida* and is the primary target of fluconazole. Studies have shown that specific mutations in the *ERG11* gene, particularly the Y132F and Y132H variants, significantly alter the structure and function of the CYP51 enzyme, reducing the binding efficiency of fluconazole to the enzyme, thereby enhancing the resistance of *Candida* to fluconazole. Additionally, molecular docking and simulation techniques have revealed that variations or conformational changes in other key residues of the CYP51 enzyme (e.g., I304, G308, I379) also affect the binding affinity, further weakening the antimicrobial efficacy (Medeiros et al., 2022). In addition to ERG11 mutations, overexpression of transporters like CDR1 and CDR2 has been confirmed to contribute to fluconazole resistance through real-time PCR and MALDI-TOF mass spectrometry, as it augments Candida’s efflux capacity, expelling fluconazole before it can exert its intracellular effects (Maenchantrarath et al., 2022). However, mutations in the *ERG11* gene and overexpression of specific transporters are not the sole mechanisms of azole resistance. In addition to these two mechanisms, other resistance mechanisms exist, including genomic amplification of the *ERG11* gene (Hu et al., 2023), mutations in transcription factors (such as TAC1, leading to overexpression of both the *ERG11* gene and efflux pump genes) (Fan et al., 2023), biofilm formation (Siqueira et al., 2025), and mutations in other genes (such as *MRR1*) that may also be associated with azole resistance (Branco et al., 2022). In summary, the resistance of *Candida* to azole drugs is the result of the synergistic action of the aforementioned multiple molecular mechanisms, which makes its treatment challenging.

#### 4.1.2 Polyene resistance

Polyene antifungals primarily include Amphotericin B and Nystatin, which mainly act by binding to ergosterol in the fungal cell membrane, leading to increased membrane permeability and subsequent leakage of cellular contents, ultimately resulting in cell death (Wang et al., 2021). Although Amphotericin B is a broad-spectrum antifungal agent, it has faced a significant challenge in clinical practice due to the emergence of resistance among *Candida* species.

The resistance mechanism of *Candida* to amphotericin B involves not only changes in cell membrane components but is also closely related to biofilm formation. In particular, biofilm formation can significantly enhance the drug resistance of *Candida*. Moreover, the *FLO8* gene, a key transcription factor in biofilm formation, plays a pivotal role in this process. Mutations in the FLO8 gene promote biofilm formation, thus significantly enhancing *Candida*’s resistance to amphotericin B (Misas et al., 2019). Therefore, in species such as *C. auris* and *C. albicans*, *FLO8* gene mutations play a pivotal role in the development of amphotericin B resistance (Fenton and John, 2024; Jin et al., 2023).

Beyond biofilm formation, variations in multiple genes associated with the ergosterol biosynthesis pathway, such as *ERG1*, *ERG2*, *ERG3*, *ERG5*, *ERG6*, *ERG11*, and *ERG13*, greatly influence *Candida*’s resistance to amphotericin B. Overexpression or functional alterations in these genes can lead to adjustments in the lipid composition of the cell membrane, thereby affecting the binding capacity of amphotericin B to the cell membrane (Chybowska et al., 2020; Sarma and Upadhyay, 2017; Iguchi et al., 2019; Chatterjee et al., 2015; Arendrup et al., 2017; Kean and Ramage, 2019). These processes involve changes in multiple genes and cellular structures, which collectively enable *Candida* to effectively resist the antimicrobial effects of amphotericin B (Bohner et al., 2022; Chaabane et al., 2019).

#### 4.1.3 Echinocandin resistance

Echinocandins, a class of antifungal agents that target the synthesis of *β*-1,3-D-glucan in the fungal cell wall (Szymański et al., 2022), inhibit the synthesis of the fungal cell wall, leading to cell death. In recent years, echinocandins have shown significant therapeutic potential in clinical treatment. These drugs are not only used independently for antifungal therapy but are also frequently combined with other antifungal or non-antifungal drugs to enhance therapeutic outcomes (Dos Reis et al., 2023). However, with the widespread use of echinocandins, resistance among *Candida* species has become a growing concern, posing a major challenge in clinical treatment.

Studies have shown that *Candida* resistance to echinocandins is predominantly mediated through mutations in the *FKS1* gene, which encodes a key enzyme in β-1,3-D-glucan synthesis (Frías-De-León et al., 2020; Lara-Aguilar et al., 2021; Kordalewska et al., 2023). As early as 2018, Kordalewska et al. reported that four *Candida* isolates from India exhibited resistance to all echinocandins tested, with minimum inhibitory concentration (MIC) values reaching or exceeding 4 mg/L within 24 h. Further analysis through PCR amplification and sequencing revealed specific mutations (e.g., S639F) in the *FKS1* gene of these resistant strains, which were absent in sensitive strains and aligned with known echinocandin resistance mechanisms. Mouse model experiments corroborated these findings, showing that strains carrying specific *FKS1* gene mutations exhibited resistance to echinocandins *in vivo*, whereas wild-type strains did not (Kordalewska et al., 2018).

Although the role of the *FKS2* gene in *Candida* drug resistance is relatively minor, studies have revealed that its mutations may also lead to partial echinocandin resistance (Asadzadeh et al., 2022). It is important to note that research on specific types, frequencies, and contributions of *FKS2* gene mutations to drug resistance remains limited and requires further in-depth investigation. *Candida* resistance to echinocandins not only complicates treatment but also significantly increases its pathogenicity and risk of infection, particularly in immunosuppressed patients (Lakshmi et al., 2018).

#### 4.1.4 5-Fluorocytosine

5-Fluorocytosine (5-FC) can be utilized to target and disrupt nucleic acid biosynthesis within cells, functioning as a nucleoside analog. When combined with polyene antifungal agents such as amphotericin B, it is a reliable option for the treatment of refractory *Candida* infections and *Cryptococcus meningitis*. This drug demonstrates good efficacy against several major *Candida* species, including *C. albicans*, *C. parapsilosis*, and *C. dubliniensis*.

The mechanisms of *Candida* resistance to 5-FC involve multiple complex levels (Czajka et al., 2023; Delma et al., 2021). Specifically, 5-FC must be metabolized intracellularly to 5-fluorouracil (5-FU) to interfere with fungal RNA and DNA synthesis. However, mutations in 5-FC metabolism-related genes (e.g., the *FUR1* gene) can impede this effective conversion, leading to resistance (Edlind Thomas and Katiyar Santosh, 2010). Additionally, alterations in cell membrane transporters, including (e.g., FCY2 and AFR1), can reduce intracellular 5-FC concentrations, while enhanced intracellular antioxidant mechanisms can better counteract the oxidative stress generated by 5-FU (Billmyre et al., 2020; Chang et al., 2021), both of which contribute to resistance development. Furthermore, changes in cell wall components, acting as the first barrier for 5-FC entry, can decrease 5-FC permeability, further contributing to resistance (Czajka et al., 2023).

In summary, the resistance mechanisms of *Candida* to antifungal drugs are multifaceted, involving gene mutations, drug transport, and cell wall synthesis (Table 1). These mechanisms not only complicate treatment but also enhance the pathogenicity and infection risk of *Candida*, posing significant challenges to clinical therapy. In this context, whole-genome studies, as a powerful tool, play an invaluable role in advancing the understanding of *Candida* resistance mechanisms, the development of novel antifungal drugs, and the innovation of therapeutic strategies. By conducting in-depth whole-genome studies and integrating advanced technologies such as high-throughput sequencing and bioinformatics analysis, it is crucial for elucidating *Candida* resistance mechanisms, developing new antifungal drugs and therapeutic strategies, and ultimately controlling *Candida* infections. This will equip clinicians and researchers with more powerful tools to confront the growing challenges posed by *Candida* infections and improve patient outcomes.

### 4.2 Mechanisms of drug resistance based on genomic variation and evolution

#### 4.2.1 Evolution and genomic variation of *Candida*

*Candida* has undergone complex evolutionary mechanisms in diverse environments, including gene flow, hybridization, and microevolution. Gene flow is a critical evolutionary force in populations of the *Candida* genus, significantly shaping the diversity and adaptability of these fungi. Specifically, *Candida* species have the ability to acquire new traits through the biological process of horizontal gene transfer (HGT) (Chakrabarti and Sood, 2021; Sekizuka et al., 2019). This genetic exchange across strains or species not only provides *Candida* with valuable genetic resources but also greatly enhances its survival and reproduction under varying environmental conditions. HGT enables *Candida* to rapidly adapt to environmental shifts, including adjustments in metabolic pathways and morphological transformations, thereby allowing *Candida* to thrive in a variety of ecological niches, including those that were not part of its original habitats (Santana et al., 2024; Rhodes et al., 2024; Santana and O'Meara, 2021). For example, *Candida* species may have initially evolved in an intermediate host, such as birds, and later acquired traits through HGT that enabled them to adapt to human habitats (Chakrabarti and Sood, 2021), thereby facilitating their spread into human populations. Additionally, due to restricted gene flow within populations, *Candida* may have developed unique adaptive traits in different regions (Chow et al., 2020). This interplay between geographic isolation and restricted gene flow further drives the diversity and geographical specialization of the *Candida* genus. Therefore, horizontal gene transfer not only provides *Candida* with a rich source of genetic variation but also promotes its rapid adaptation and dissemination in different environments, making it a globally emerging multidrug-resistant fungal pathogen (Santana et al., 2024; Yue et al., 2018; Santana and O'Meara, 2021). In recent years, genomic sequencing, comparative analysis, and gene mutation detection have enabled scientists to identify the primary sources of *Candida* drug resistance. Ksiezopolska and Gabaldón, (2018) identified that the causes of resistance to major antifungal drugs, such as fluconazole and amphotericin B, are often due to mutations in specific genes (Ksiezopolska and Gabaldón, 2018). These mutated genes, such as *Erg11*, *Upc2p*, *Mrr1p*, *Tac1p*, *CDR1*, and *CDR2*, are particularly prevalent in specific *Candida* populations (Lohberger et al., 2014; Znaidi et al., 2008; Wang et al., 2015). The distribution of these genetic variations among different populations is closely related to the genetic background of the populations, the environmental pressures, and the selective pressures for drug resistance.

Microevolutionary processes also play a critical role in the evolution of *Candid*a. Selective pressures in different environments drive genetic mutations and selective amplification in *Candida*, leading to significant changes in its survival and pathogenic capabilities. Studies have shown that *Candida* can rapidly develop drug resistance through microevolution when exposed to antifungal drugs, an adaptive mechanism that enhances its survival in clinical settings (Kannan et al., 2019). For example, research on *C. glabrata* has revealed that this species achieves adaptation to antifungal drugs through small-scale genomic changes, such as copy number variations (CNVs) (Ropars et al., 2018). Further studies indicate a close link between drug resistance and adaptability in *Candida*. Research on *C. albicans* has shown that certain drug-resistant mutations not only enhance the survival of *Candid*a in drug-exposed environments but are also associated with increased virulence traits. This association may involve multiple levels of biological processes, including genomic instability, altered metabolic activity, and gene recombination. These processes are driven not only by genetic mutations but may also be influenced by the interaction of environmental and genetic factors (Priest et al., 2020). These physiological and biochemical changes, whether acting independently or synergistically, significantly enhance the adaptability and pathogenicity of *Candida*, making it more difficult to control infections.

Collectively, the evolutionary mechanisms of *Candida* in different environments are multifaceted and complex, with gene flow, hybridization, and microevolution collectively driving the development of its adaptability and pathogenicity. The variations resulting from these evolutionary mechanisms, such as point mutations, insertions/deletions, and CNVs, are pivotal in the development of antifungal resistance in *Candida*, providing valuable biological insights for the control of *Candida* infections.

#### 4.2.2 Point mutations

During the process of *Candida* developing resistance to antifungal drugs, point mutations serve as a core mechanism, playing a crucial role (Table 2). Specifically, regarding the antifungal drug fluconazole, studies have revealed that point mutations in the *ERG11* gene are one of the key factors leading to resistance. These genetic alterations modify the drug’s binding site, diminishing the affinity between fluconazole and its molecular target, thereby enabling *Candida* to circumvent the drug’s antimicrobial action (Medeiros et al., 2022). Similarly, echinocandin resistance has been widely reported as being associated with mutations in the *FKS1* gene, which are recognized as an important resistance mechanism. These mutations not only affect the interaction between echinocandins and the target enzyme but may also induce functional changes in the enzyme, endowing *Candida* with resistance to echinocandins (Lara-Aguilar et al., 2021; Kordalewska et al., 2023). Drug resistance in *Candida* is not only due to genetic mutations but can also spread through genetic recombination and gene flow among distinct species. Research by Märtson et al. (2021) has shown that certain *Candida* species, such as *C. albicans* and *C. glabrata*, can develop resistance to multiple antifungal drugs. These resistance genes can be transmitted through genetic recombination and gene flow among different species, leading to an increase in resistant strains (Märtson et al., 2021). Moreover, some non-*Candida albicans* species, such as *C. krusei* and *C. tropicalis*, are inherently more resistant to certain antifungal drugs, which also complicates therapeutic strategies.

**Table 2:** The relationship between *Candida*’s gene/protein mutations and antifungal drug resistance.^#^

Gene/protein	Primary mutations	Primary mutation types	Antifungal resistance	*Candida* species	Primary resistance mechanisms
ERG11 (lanosterol 14a-demethylase)	S405F, G464S, G448E, F449S, A61V, R467K and I471T	Non-synonymous substitution	Fluconazole	*C. albicans*	Modifying the structure and function of CYP51 enzyme reduces the binding efficiency of drugs to CYP51, thereby enhancing the drug resistance of *Candida* (Zhang et al., 2019).
	K143R, Y132H, Y132F and K143Q	Non-synonymous substitution	Fluconazole and voriconazole		
	A114S and Y257H	Non-synonymous substitution	Fluconazole and voriconazole		
	Y18F, T315A, Y118A and Y118T	Non-synonymous substitution	Fluconazole		
	F126T, Y132F and K143R	Non-synonymous substitution	Fluconazole	*C. auris*	
	Y132F	Missense	Fluconazole	*C. tropicalis*	
	K143R	Non-synonymous substitution	Fluconazole, voriconazole and itraconazole		
	Y166S	Non-synonymous substitution	Voriconazole	*C. krusei*	
ERG3 (C5 sterol desaturase)	Q139A	Non-synonymous substitution	Fluconazole	*C. glabrata*	The *ERG3* gene encodes C-5 sterol dehydrogenase. When this gene is inactivated, *Candida* can bypass the normal sterol biosynthesis pathway, thereby developing drug resistance (Branco et al., 2017).
UPC2 (TF, regulates most ERG genes)	A643T, Y642F, G648D, G648S, A646V and W478C	GOF substitution	Fluconazole	*C. albicans*	The UPC2 transcription factor enhances the drug resistance of *Candida albicans* to antifungal agents by modulating the expression of *ERG* genes, which influences the content and composition of sterols in the cell membrane (Dunkel et al., 2008).
	A643V	GOF substitution	Fluconazole		
	G307S and G448E	GOF substitution	Fluconazole		
CDR1 + CDR2 (ABC-Ts)	Chr 3 trisomy	Increased cdr1 and cdr2 copy numbers	Azole	*C. albicans*	CDR1 and CDR2, two ABC transporters, enhance extracellular efflux capability, reduce intracellular drug accumulation, and act in concert with other resistance mechanisms to confer multidrug resistance (Prasad et al., 2015).
TAC1 (TF, regulates CDR1, CDR2 and PDR16)	V736A, N972D, T225A, N977D, G980E and G980W	GOF substitution	Azole	*C. albicans*	Enhances efflux capability (*CDR1*, *CDR2*), alters cell membrane permeability (*PDR16*), and can synergize with other resistance mechanisms, leading to increased drug resistance (Saidane et al., 2006).
	F214S, K143R, R495G and A640V	Non-synonymous substitution	Fluconazole	*C. auris*	
MRR1 (TF, regulates MDR1)	P683S and P683H	GOF substitution	Azole	*C. albicans*	By upregulating the expression of the *MDR1* multidrug resistance transporter, the cellular efflux capacity is enhanced, thereby increasing drug resistance (Fei et al., 2020).
	T374I, S595Y and C866Y	GOF substitution	Azole	*C. dubliniensis*	
	T965△ and (D987-I998)△	Deletion	Azole		
PDR16 (phosphatidylinositol transfer protein)	△pdr16	Deletion	Reduced resistance to fluconazole, itraconazole and ketoconazole miconazole	*C. glabrata*	PDR16 enhances the barrier function of the cell membrane by modulating the composition of phospholipids in the cell membrane, and acts in concert with other resistance mechanisms to confer multidrug resistance (Cohen, 2014).
PDR1 (TF, regulates CDR1, SNQ2, PDH1 and QDR2)	D261G, R293I, R592S, G583S, Y584C, D876Y, L280F, N691D, L328F, R376W, D1082G, T588A, T607S, E1083Q, S316I, S343F and R376G	GOF substitution	Fluconazole	*C. glabrata*	PDR1 enhances the cell membrane barrier function by regulating the expression of multidrug resistance transporters and collaborates with other resistance mechanisms, leading to the development of multidrug resistance (Cavalheiro et al., 2018).
FKS1 ( 1–3 glucan synthase)	S645F	Non-synonymous substitution	Echinocandin	*C. albicans*	The *FKS1* gene encodes 1,3-*β*-*D*-glucan synthase, which plays a critical role in the synthesis and maintenance of the cell wall in *Candida* and is a target for multiple antifungal drugs. Variations in or upregulation of this gene can lead to enhanced cell wall synthesis, thereby conferring drug resistance (Yang et al., 2017).
	F635L, F635Y, S639F and R1354S	Non-synonymous substitution	Echinocandin	*C. auris*	
	F625C and S629P	Non-synonymous substitution	Echinocandin	*C. glabrata*	
	F625△	Deletion	Echinocandin		
	F655C	Non-synonymous substitution	Echinocandin	*C. krusei*	
	P660A	Non-synonymous substitution	Echinocandin	*C. parapsilosis*	
FKS2 ( 1–3 glucan synthase)	F659S and F659V	Non-synonymous substitution	Echinocandin	*C. glabrata*	Variations or upregulated expression of the *FKS2* gene can lead to enhanced cell wall synthesis, thereby conferring drug resistance (Rodrigues and Henriques, 2018).
	F659△	Deletion	Echinocandin		
	S663P and S663F	Non-synonymous substitution	Echinocandin		
	E655K, E655G, P667H and P667T	Non-synonymous substitution	Echinocandin		
	R1378S and R1378G	Non-synonymous substitution	Echinocandin		
FCA1/FCY1 (cytosine deaminase)	G28D and S29L	LOF substitution	5-FC	*C. albicans*	The *FCA1*/*FCY1* gene encodes a cellular cytidine deaminase that activates certain antifungal drugs in *Candida*. Downregulation or deletion of this gene leads to the inactivation of these antifungal drugs, resulting in drug resistance (Edlind, 2011).
	S29L	Non-synonymous substitution	5-FC	*C. dubliniensis*	
	A15D, G11D and W148R	Non-synonymous substitution	5-FC	*C. glabrata*	
FCY2 (cytosine permease)	A176G	LOF substitution	5-FC	*C. albicans*	The *FCY2* gene encodes a cellular cytidine transporter that facilitates the transport of the antifungal drug 5-FC into *Candida* cells. If the expression of this gene is downregulated or absent, 5-FC cannot enter the cell, leading to drug resistance (Delma et al., 2021).
	G145T	Non-synonymous substitution	5-FC	*C. tropicalis*	
FUR1 (uracil phosphoribosyl transferase (UPRT))	C101R	LOF substitution	5-FC	*C. albicans*	The *FUR1* gene encodes uracil phosphoribosyl transferase, which plays a role in the metabolic activation of the antifungal drug 5-FU in *Candida*. If the expression of this gene is downregulated or absent, 5-FU cannot be converted to its active form, leading to drug resistance (Vandeputte et al., 2011).
	G190D	LOF substitution	5-FC	*C. glabrata*	
	I83K and D193G	LOF substitution	5-FC/5-FU		
	△G73-V81	LOF Deletion	5-FC/5-FU		
MSH2 (DNA mismatch repair)	V239L	Non-synonymous substitution	Fluconazole or echinocandin	*C. glabrata*	Defects in the *MSH2* gene promote the generation of drug-resistant genes by increasing genomic instability and mutation rates in *Candida*. However, the specific drug-resistant phenotype requires analysis in conjunction with other factors (Bordallo-Cardona et al., 2019).
FLO8 (transcription factor)	Chr 3 trisomy	LOF Deletion	Amphotericin B or fluconazole	*C. glabrata*	The FLO8 transcription factor is a crucial regulator of *Candida* drug resistance mechanisms, influencing *Candida*’s drug resistance by modulating processes such as cell morphogenesis, adhesion capabilities, and expression of drug resistance genes (Cao et al., 2006).
	G723R and T751D	Non-synonymous substitution	Enhancement of toxicity		Enhanced activity of the Flo8 C-terminus (Liu et al., 2015), thereby promoting fungal filamentous growth and increasing its pathogenicity (Bester et al., 2006; Cao et al., 2006).

^#^The partial content of the above table is mainly referenced from the report by Czajka et al. (2023). LOF, loss of function; GOF, gain of function.

Therefore, point mutations play a crucial role in the development of antifungal drug resistance in *Candida*, whether against fluconazole or echinocandins. Mutations in key genes like *ERG11* and *FKS1* are critical in leading to treatment failure. Currently, point mutations at the gene level primarily include non-synonymous substitutions, missense mutations, and deletions; at the amino acid level, they can be categorized into gain-of-function (GOF) substitutions and loss-of-function (LOF) substitutions (Chaabane et al., 2019; Frías-De-León et al., 2020; Table 1).

#### 4.2.3 Copy number variation

Copy Number Variation (CNVs) refers to changes in the copy number of certain genes within the genome, a mechanism that is also commonly observed in the development of antifungal resistance in *Candida* (Table 2). Specifically, studies have found that upon exposure to antifungal drugs, the copy number of genes associated with drug transporters in *Candida*’s genome significantly increases (Maenchantrarath et al., 2022). This increase in copy number directly enhances the capacity of *Candida* to expel the drug, thereby reducing the effective intracellular drug concentration and enhancing the organism’s resistance. Furthermore, as *Candida* continuously adapts to antifungal drug pressure, its genome undergoes a series of changes, including the emergence of numerous new high-copy-number genes (Todd and Selmecki, 2020). These newly emerged CNVs not only endow *Candida* with the ability to rapidly adapt to drug environments but also enable it to survive and reproduce under drug pressure. Therefore, CNVs play a significant role in the development and maintenance of antifungal resistance in *Candida*.

#### 4.2.4 Microevolution

Due to the limited gene flow between different geographic regions, unique resistant genotypes can emerge in localized areas (Chow et al., 2020). This genetic differentiation, driven by geographic isolation, forms a complex foundation for the evolution of antifungal resistance in *Candida*. Additionally, *Candida* also has the ability to acquire novel resistance genes through horizontal gene transfer (HGT). This process is further enhanced by the hybridization between different genotypes of *Candida*, significantly facilitating the rapid formation and extensive dissemination of genetic diversity (Chakrabarti and Sood, 2021; Gifford et al., 2024). The rapid diversification of its genome, particularly linked to the rapid development of resistance (Bravo Ruiz et al., 2019; Wang and Xu, 2022), highlighting the high adaptability and flexibility of *Candida* in response to various clinical treatment challenges. In light of the growing challenge of antifungal treatment for *Candida*, it is important to enhance the monitoring and management of its resistance to curb further spread (Ionescu et al., 2024). To address this global issue, researchers are vigorously exploring new therapeutic approaches, including the development of monoclonal antibodies targeting *Candida* (Singh et al., 2023), aiming to break the limitations of traditional treatments. Effectively addressing the multidrug resistance of *Candida* is crucial for enhancing the efficacy of clinical treatment and protecting patients from the threat posed by these pathogens.

In conclusion, the drug resistance of *Candida* is a complex and multifactorial process involving genomic variations, evolutionary mechanisms, and microevolutionary processes. Genetic variations such as point mutations, CNVs, and gene flow play pivotal roles in the development and dissemination of resistance. These mechanisms not only affect the sensitivity of *Candida* to existing antifungal drugs but also pose challenges for the development of novel drugs and therapeutic strategies. Consequently, a comprehensive understanding of the molecular mechanisms of *Candida* drug resistance is essential for developing effective preventive measures and innovative treatment methods. In the future, it is essential to further strengthen resistance surveillance, explore new therapeutic targets, and promote interdisciplinary collaboration to effectively address this growing global health issue.

## 5 Future directions

In summary, the field of genomics has witnessed remarkable advancements in understanding *Candida* infections over recent years. These findings have provided new insights into the complex biological behavior of *Candida*, enhancing our understanding of its virulence and resistance to antifungal agents, and informing the development of novel therapeutics and public health strategies. However, the pathogenicity and drug resistance of *Candida* remain complex and dynamic issues that require ongoing research and exploration. Future research should delve into the genomic resources and genetic diversity of *Candida* to uncover additional genes and novel mechanisms related to pathogenicity and drug resistance. Moreover, integrating clinical data with experimental validation is imperative for devising innovative treatment modalities and preventive measures to counter the public health threat posed by *Candida* infections.

**Figure 5:**
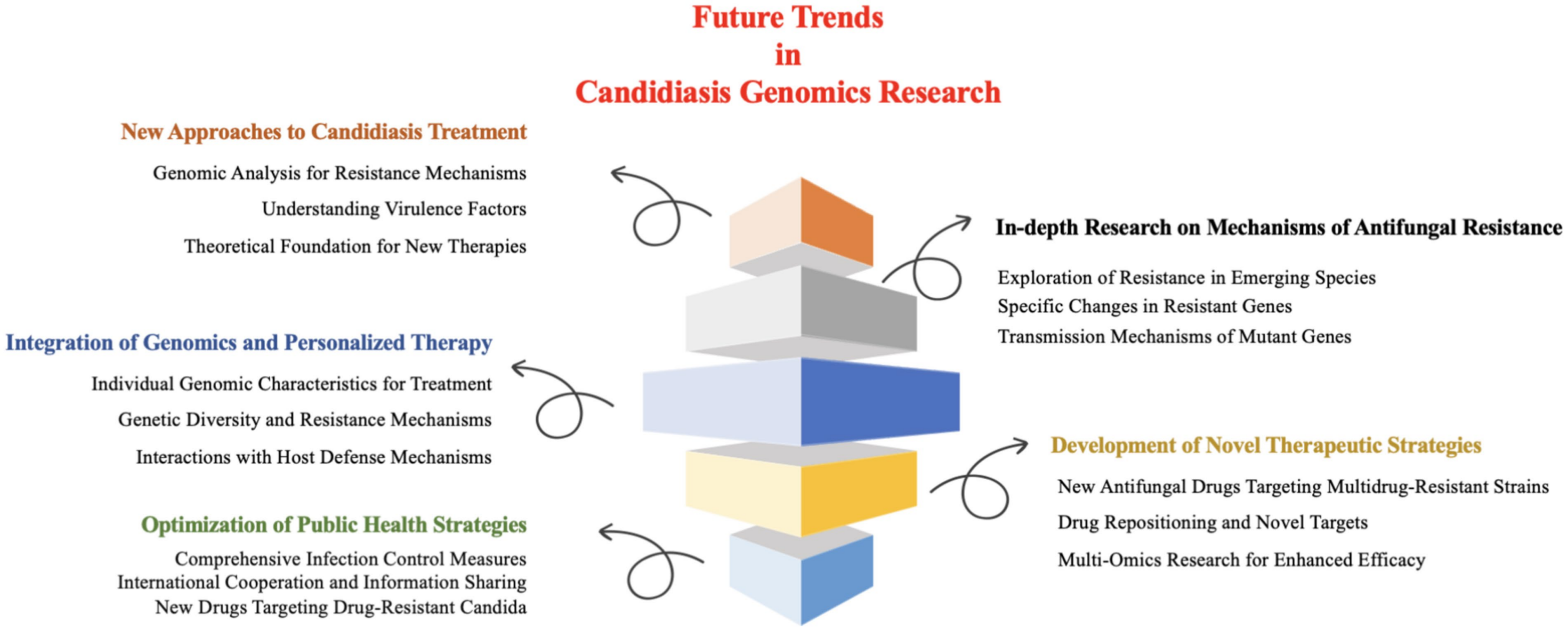
Future directions in *Candida* genomics research.

Collectively, future research on *Candida* infections should prioritize the following key issues and research directions (Figure 5).

### 5.1 New approaches to candidiasis treatment: the significance of genomics research

Currently, the treatment strategies for candidiasis predominantly focus on the use of antifungal drugs, but the issue of drug resistance is becoming increasingly severe, which imposes a significant medical and economic burden. Therefore, it is crucial to explore new treatment methods and preventive measures. Genomics has shown broad application prospects in candidiasis research and offers new avenues and methods for its research and treatment (Lass-Flörl et al., 2024; Rhodes and Fisher, 2019). By conducting in-depth analysis of the *Candida* genome, researchers can uncover resistance mechanisms, achieve a better understanding of their virulence factors and regulatory mechanisms, providing a theoretical foundation for the development of new diagnostic and therapeutic strategies (Rhodes and Fisher, 2019; Fidel, 2011; Rybak et al., 2022; Chen et al., 2024). These research findings not only enhance the overall understanding of candidiasis but also promote the development of precision medicine, which could help alleviate and address the pressures and challenges of related public health issues. These research advances provide theoretical support for developing new therapeutic strategies, while specific treatment methods are continually being explored.

Based on the achievements of genomic research, investigators are exploring a multitude of novel therapeutic strategies. Alternative therapies for *Candida* infections and biofilm disruption strategies offer new perspectives for clinical treatment. Research has found that natural plants such as sesame, sesame leaves, and honeysuckle contain antifungal components that can be used to treat oral candidiasis. They are being explored as alternative options for antifungal drugs, capable of inhibiting the growth of oral *Candida* (Contaldo et al., 2023). Meanwhile, probiotic therapy, particularly strains such as *Lactobacillus* sp. and *Bifidobacterium* sp., effectively prevents the recurrence of *Candida* infections on oral mucosa and skin surfaces by restoring microbial balance at the infection sites. With low toxicity, it offers a safer treatment option in clinical settings (Contaldo, 2023; Fusco et al., 2023; Ricci et al., 2022). Moreover, photodynamic therapy utilizes photosensitizers and specific wavelengths of light to selectively destroy *Candida* cells. This method offers advantages such as non-induction of drug resistance, high selectivity, and low toxicity, introducing a novel physical approach for the treatment of *Candida* infections (Contaldo et al., 2023). Additionally, biofilm degradation strategies employ methods such as enzymes (like DNase) and metal chelators to break down fungal biofilms associated with *Candida* infections. This indirectly inhibits fungal growth and is particularly suitable for cases of recurrent or refractory *Candida* infections, providing a new approach to enhance the efficacy of antifungal drugs and reduce infection recurrence rates (Contaldo et al., 2023; Contaldo, 2023; Fusco et al., 2023; Chanda et al., 2017). These strategies and research prospects have opened new avenues for the clinical treatment of candidiasis infections, warranting further in-depth investigation and exploration.

### 5.2 In-depth research on mechanisms of antifungal resistance

Despite considerable research on antifungal resistance, further exploration is needed to understand how different *Candida* species develop resistance in various environments. Particularly, the complexity and severity of resistance in emerging species or strains, such as *C. auris*, have garnered significant attention (Rybak et al., 2022). *C. auris* exhibits multidrug resistance, showing resistance to commonly used antifungal agents like polyenes, echinocandins, and 5-FC (Bhattacharya et al., 2020; Rybak et al., 2022). Therefore, in-depth investigation into the specific changes in resistant genes during the mutation process and the transmission of these mutant genes within *Candida* populations has become a current research focus. However, existing research still faces some challenges and limitations. For example, in-depth studies on the *Candida* resistance mechanisms require high-precision experimental techniques and complex data analysis methods (Branco et al., 2023), demanding high levels of expertise and experimental conditions. Additionally, *Candida* resistance mechanisms may be influenced by multiple factors, including environmental factors and host immune status (Costa-de-Oliveira and Rodrigues, 2020; Czajka et al., 2023), and the interactions between these factors make the research even more complicated. To address these challenges, future research could explore the following aspects: First, continue to deepen the fundamental research on the mechanisms of *Candida* drug resistance mechanisms to uncover additional unknown resistance mechanisms; Second, strengthen interdisciplinary collaboration and utilize advanced technologies, such as bioinformatics and genomics, to conduct more comprehensive analysis and prediction of *Candida* drug resistance; Finally, foster the innovative development of antifungal drugs and therapeutic strategies to provide more effective solutions for combating the threat of emerging drug-resistant *Candida*. In particular, systematic research on the variation and transmission mechanisms of resistance genes in emerging drug-resistant species or strains, such as *C. auris*, and the formulation of targeted treatment strategies will be the top priority for future research (Jangir et al., 2023). Only through in-depth research and continuous exploration can we better understand and address the challenges of *Candida* drug resistance, ensuring the safeguarding of human health.

### 5.3 Integration of genomics and personalized therapy

With the rapid advancement of genomics technology, we are entering a new era where personalized treatment of *Candida* infections based on individual genomic characteristics is becoming feasible. Currently, in-depth analysis of the genomic data of approximately 2,000 primary *Candida* species (Schikora-Tamarit and Gabaldón, 2024), alongside genomic studies of emerging pathogens such as *C. albicans* (Bruno et al., 2021), has shed light on the genetic diversity and resistance mechanisms of *Candida*. Additionally, the integration of transcriptomic analysis and functional genomics approaches has provided new insights into the interactions between *Candida* and host defense mechanisms, offering new perspectives for personalized therapy. In the future, further exploration of the relationship between *Candida* resistance genomics and clinical treatment responses is expected to provide a scientific basis for personalized medicine (Stalder et al., 2023). However, achieving personalized treatment for *Candida* infections necessitates a holistic examination of pharmacokinetics and pharmacogenomics, and other pertinent factors (Daneshnia et al., 2023). Interdisciplinary collaboration and resource integration will be key to advancing this field.

### 5.4 Development of novel therapeutic strategies

Attention should be focused on the development of new antifungal drugs, particularly those targeting multidrug-resistant *Candida* strains, which is essential for improving treatment outcomes. With the continuous advancement of genomics technology, personalized treatment based on the individual genomic characteristics of *Candida* may become a reality, laying the foundation for personalized medicine. To effectively address the challenge of multidrug-resistant *Candida*, researchers are actively employing strategies such as drug repositioning, identification of novel targets, and the discovery or synthesis of novel compounds to explore novel antifungal drugs that can effectively inhibit these resistant strains. Meanwhile, by integrating multi-omics research methods, including transcriptomics and metabolomics, new drug targets and combination therapy strategies are being further explored to enhance treatment efficacy (Brackin et al., 2021). For example, *Candida* biofilms have been identified as a promising target (Kovács et al., 2021; Jamiu et al., 2021), and combination therapies may potentially overcome their resistance in the future. Collectively, the development of new antifungal drugs in combination with multi-omics research methods will be an important and effective strategy for improving the treatment outcomes of multidrug-resistant *Candida*.

### 5.5 Optimization of public health strategies

Considering the transmission characteristics of *Candida*, comprehensive and in-depth measures are imperative to address the public health challenges posed by *Candida* infections. Strengthening public health surveillance, particularly infection control in hospital environments, should be a priority. This includes implementing strict contact isolation policies, ensuring thorough environmental cleaning and disinfection to curb the spread of *Candida* within hospitals. Additionally, promoting international cooperation to facilitate information sharing and coordinated responses on a global scale is crucial for this cross-border challenge (Rhodes and Fisher, 2019). Close collaboration between public health departments and healthcare institutions is also indispensable, as each brings its own expertise in areas such as surveillance and early warning, the development of prevention and control strategies, and patient diagnosis and treatment. Such collaborative efforts can markedly enhance the effectiveness of control measures (Borman and Johnson, 2020). The development of new drugs targeting drug-resistant *Candida* is urgent, utilizing multi-omics research methods to explore new drug targets and combination therapy strategies will open new avenues for treating drug-resistant *Candida* infections (Janniger and Kapila, 2021).

In summary, comprehensive strengthening of surveillance, international cooperation, hospital infection control, inter-departmental collaboration, and new drug development are key strategies for addressing the public health issue of *Candida* infections. Through in-depth exploration of these research directions, a better understanding of the pathogenic mechanisms and drug resistance of *Candida* infections can be achieved, ultimately facilitating the development of more effective prevention and treatment strategies.
